# Choroid Sprouting Assay: An *Ex Vivo* Model of Microvascular Angiogenesis

**DOI:** 10.1371/journal.pone.0069552

**Published:** 2013-07-26

**Authors:** Zhuo Shao, Mollie Friedlander, Christian G. Hurst, Zhenghao Cui, Dorothy T. Pei, Lucy P. Evans, Aimee M. Juan, Houda Tahir, François Duhamel, Jing Chen, Przemyslaw Sapieha, Sylvain Chemtob, Jean-Sébastien Joyal, Lois E. H. Smith

**Affiliations:** 1 Department of Ophthalmology, Boston Children's Hospital, Harvard Medical School, Boston, Massachusetts, United States of America; 2 Department of Ophthalmology, Research Centers of Hôpital Maisonneuve-Rosemont, University of Montreal, Montreal, Quebec, Canada; 3 Departments of Pediatrics Ophthalmology and Pharmacology, Research Centers of CHU Sainte-Justine, Montreal, Quebec, Canada; Institut de la Vision, France

## Abstract

Angiogenesis of the microvasculature is central to the etiology of many diseases including proliferative retinopathy, age-related macular degeneration and cancer. A mouse model of microvascular angiogenesis would be very valuable and enable access to a wide range of genetically manipulated tissues that closely approximate small blood vessel growth *in vivo*. Vascular endothelial cells cultured *in vitro* are widely used, however, isolating pure vascular murine endothelial cells is technically challenging. A microvascular mouse explant model that is robust, quantitative and can be reproduced without difficulty would overcome these limitations. Here we characterized and optimized for reproducibility an organotypic microvascular angiogenesis mouse and rat model from the choroid, a microvascular bed in the posterior of eye. The choroidal tissues from C57BL/6J and 129S6/SvEvTac mice and Sprague Dawley rats were isolated and incubated in Matrigel. Vascular sprouting was comparable between choroid samples obtained from different animals of the same genetic background. The sprouting area, normalized to controls, was highly reproducible between independent experiments. We developed a semi-automated macro in ImageJ software to allow for more efficient quantification of sprouting area. Isolated choroid explants responded to manipulation of the external environment while maintaining the local interactions of endothelial cells with neighboring cells, including pericytes and macrophages as evidenced by immunohistochemistry and fluorescence-activated cell sorting (FACS) analysis. This reproducible *ex vivo* angiogenesis assay can be used to evaluate angiogenic potential of pharmacologic compounds on microvessels and can take advantage of genetically manipulated mouse tissue for microvascular disease research.

## Introduction

Dysregulation of angiogenesis in microvessels is associated with many sight-threatening vascular eye diseases such as diabetic retinopathy (DR)[1], retinopathy of prematurity (ROP)[2] and age-related macular degeneration (AMD)[3]. In addition, there are many other diseases that have a microvascular component including cancer[4], rheumatoid arthritis[5], psoriasis and the healing process after stroke[6]. The development of an efficient and reproducible assay modeling the microvascular bed would enhance our ability to study microvascular diseases. Current microangiogenic models using endothelial cells (ECs) in culture are very useful but are limited by the lack of interactions with other cells that are involved in angiogenesis, such as pericytes and macrophages. These models are also hindered by the difficulty in isolating mouse ECs, which might provide genetically altered cells for study. The aortic ring assay in which isolated murine aortas are cultured is very helpful for studying large vessel sprouting but may not represent microvascular angiogenesis. To overcome these limitations, we developed a sprouting model using choroidal tissue that can easily be isolated from genetically modified mice to explore microvascular angiogenesis.

The choroid is a vascular bed in the eye beneath the retinal pigment epithelium (RPE) and supplies oxygen and nutrients to the outer retina. It comprises a unique capillary meshwork with the highest blood flow in the body[7]. The integrity of the choroidal vasculature is vital to vision and abnormal angiogenesis in the choroid is associated with ocular diseases such as age-related macular degeneration (AMD)[3], retinitis pigmentosa (PR)[8], diabetic retinopathy (DR)[9] and oxygen-induced retinopathy (OIR)[10]. Thus the choroidal microvascular tissue is involved in many eye disease processes.

The choroid can be readily isolated from genetically modified transgenic mice and therefore complements *in vivo* findings for angiogenesis research. In culture the choroid rapidly develops vascular sprouts that can be quantified with high reproducibility. Our assay allows efficient evaluation of pharmacologic compounds for their angiogenic potential. It is particularly useful for AMD research since the blinding lesions of pathologic neovascularization in neovascular AMD (i.e. wet AMD) originates from the choroid.

The choroidal sprouting assay addresses an unmet need for a microangiogenesis model. It is complementary to the aortic ring assay, the most widely used organotypic model of large vessel angiogenesis. Like choroid explants, vascular sprouts from aortic explants are composed of ECs, pericytes and macrophages[11]. Aortic explant sprouting can also be manipulated by exogenous factors and the response can be quantified[12]. However, sprouts from large vessels may differ from capillary networks involved in microvascular pathology. ECs from different vasculature beds display remarkably heterogenic structure, function, protein composition and mechanism of regulation[13], [14]. For example, ECs in arteries, veins and capillaries have different tolerances to hydrostatic and oncotic pressure. Some are impermeable whereas others allow easy exchange of gas and soluble substances with extracellular fluid. Moreover, capillary ECs exhibit tissue-specific differentiation patterns. In the central nervous system (CNS), such as the brain and retina, ECs connect through tight junctions to create the blood-brain/retina barrier ensheathed by glial cells (astrocytes and Müller cells[15]). In contrast, in tissues like the kidney capillaries are fenestrated to allow renal filtration. These essential features of microvascular capillary networks may be difficult to mimic using the macrovessels of aortic rings. The choroidal sprouting assay is therefore likely to be more representative of microvascular angiogenesis, particularly of choroidal tissue and fenestrated vessels.

Other *in vitro* systems of pure endothelial cell culture have been widely used in studies of angiogenesis and enhance our understanding EC-specific signaling pathways. Cultured ECs allow controlled manipulation of the extracellular environment to investigate specific responses to cytokines, growth factors or drugs during proliferation and differentiation. However, specific inhibition of a gene of interest in EC is usually not 100% complete with small RNA interference. Furthermore, isolation and culture of pure primary microvascular ECs from any mouse including transgenic animals remain a challenge in vascular biology. Even if successfully isolated from other animals, the primary ECs differentiate after a few passages[16]. By growing ECs on extracellular matrix (Matrigel™) or gelatin, tube formation assays can provide additional information about EC function[17]. Nevertheless these vascular tubes, although responsive to common growth factors like vascular endothelial growth factor (VEGF), often lack directional organization, making quantification of these tube-like EC structures sometimes difficult[17].

Another common EC angiogenesis assay, the spheroid assay, is also cultured on extracellular matrix[18]. Multicellular EC clumps develop tube-like sprouts that project outwards from the central cellular mass with densities proportional to the rate of EC proliferation[19]. This assay of pure EC is more rigorously standardized, but also lacks the interaction of ECs with other perivascular cells and the problem of isolating ECs from genetically altered mice remains. The choroid sprouting assay was developed to overcome some of these limitations[20].

In this study, choroidal vascular tissue was isolated from different strains of mice and from rats and was cultured *ex vivo* as a model of a complex microvascular bed comprising many vascular cells with their interactions intact. We standardized this assay with respect to tissue preparation, intra- and inter- animal reproducibility, and reproducibility of pharmacological intervention on proliferation. We developed a semi-automated method for efficient and reproducible quantification of vascular sprouting in this assay. This *ex vivo* choroid angiogenic assay can be used to complement *in vivo* studies of microvascular behavior, particularly for neovascular AMD studies, and provides a tool for the assessment of pharmacologic compounds in angiogenic research.

## Materials and Methods

### Animals

Rodents used in this study were C57BL/6J (Jackson Laboratory) mice, 129S6/SvEvTac (Taconic Farms, Inc.) mice and Sprague Dawley rats (Charles River Laboratories). All studies adhered to the Association for Research in Vision and Ophthalmology Statement for the Use of Animals in Ophthalmic and Vision Research and were approved by the Animal Care Committee of Children's Hospital or Hôpital Sainte-Justine. Animals were raised in 12 hour light-dark cycles with free access to food and water.

### Dissection of choroid tissue and culture preparation

Five minutes after injecting with Avertin, animals were checked for responses and euthanized by cervical dislocation. Eyes were immediately enucleated and kept in ice-cold medium before dissection. The choice of the medium was identical to the one used for incubation later. After removing the cornea and the lens from the anterior of the eye, the central or peripheral choroid-scleral complex was separated from the retina and cut into approximately ∼2 mm×1 mm pieces (rats) or 1 mm×1 mm (mice). Choroid/sclera (here on referred to as “choroid”) fragments were isolated with and without RPE removal by peeling RPE away with forceps and placed in growth factor-reduced Matrigel™ (BD Biosciences, Cat. 354230) seeded in 24 well plates (**Supporting Video S1**). 30 µL of matrigel was used to coat the bottom of 24 well plates without touching the edge of the well. The thickness of the matrigel was approximately 0.4 mm. After seeding the choroid, plates were incubated in a 37°C cell culture incubator without medium for 10 minutes in order for the Matrigel™ to solidify. 500 µL of medium was then added to each well and incubated at 37°C with 5% CO_2_ for 48 hr before any treatment. Medium was changed every 48 hr. Phase contrast photos of individual explants were taken daily using a ZEISS Axio Oberver.Z1 microscope. The areas of sprouting were quantified with computer software ImageJ 1.46r (National Institute of Health). The macro for SWIFT-Choroid quantification is available from the authors.

### FACS cell sorting analysis

After 6 days of incubation, the Matrigel™ embedding the choroid explants was dissolved by dispase (BD Biosciences, Cat. 354235) at 37°C for 2 hours. The choroidal tissue was removed from the medium by forceps and repeated pipetting separated the sprouts. Phycoerythrin (PE) labeled anti-CD31 antibody and Alexa Fluor 488 labeled isolectin GS-IB_4_ from *Griffonia simplicifolia* (Life Technologies, Cat. I21411) were used to label EC from the sprouts. Pure Human Umbilical Vein Endothelial Cells (HUVEC; Lonza, Cat. C2519A) were used as a positive control and Human Embryonic Kidney Cells (HEK293; Sigma-Aldrich, Cat. 85120602) were used as a negative control for staining. After 4 hours of staining, samples were washed 3 times for 10 minutes each with PBS. Fluorescence intensity was monitored with a flow cytometer (BD Biosciences, Lincoln Park, NJ) and data were analyzed using the Flowjo software (Tree Star, Inc.).

### Immunohistochemistry

After 4 days of incubation, choroidal and aortic ring tissue with endothelial sproutings was washed once with ice-cold phosphate buffered saline (PBS). Paraformaldehyde (PFA) (4% solution) was then added for 10 minutes before treatment with Alexa Fluor 594 conjugated isolectin GS-IB_4_ from *Griffonia simplicifolia* (Life Technologies, Cat. I21413) and 4′, 6-diamidino- 2 phenylindole (DAPI), chondroitin sulfate proteoglycan neuron-glial antigen 2 (NG2; Chemcon, Cat. AB5320), Desmin (Abcam, Cat. Ab15200) or Alexa Fluor 488 conjugated CD68 (AbD seroTec, Cat. MCA1957A488).

### Real-time PCR

After 6 days of incubation, the sprouting cells from choroid and aortic ring explants were isolated as described for FACS analysis. mRNA of the sprouts was extracted by RNeasy® kit (Qiagen, Cat. 74104) following the manufacture's protocol. Total RNA (1–5 µg per sample) was diluted into a volume of 15 µL and incubated at 70°C for 10 minutes to dissociate the secondary structure. A master mix of 6 µL of 5× first strand buffer, 3 µL of DTT, 2 µL of dNTP, 1 µL of RNase and 1 µL of oligo-dT with 2 µL of M-MLV reverse transcription enzyme was added to the diluted RNA sample and incubated at 42°C for 60 minutes followed by 5 minutes of inactivation at 94°C. These cDNA samples were then mixed with 2× QuantiTect SYBER PCR Master Mix and primer pairs for the quantitative measurement of corresponding gene expression. Primer sequences were CD31: 5′- GAGCCCAATCACGTTTCAGTTT-3′ (forward) and 5′- TCCTTCCTGCTTCTTGCTAGCT-3′ (reverse); VE-Cadherin: 5′- TCCTCTGCATCCTCACTATCA CA -3′ (forward) and 5′- GTAAGTGACCAACTGCTCGTGAAT-3′ (reverse); NG2: 5′- GGGCTGTG CTGTCTGTTGA-3′ (forward) and 5′- TGATTCCCTTCAGGTAAGGCA-3′ (reverse); CD68: 5′- CAC CACCAGTCATGGGAATG-3′ (forward) and 5′- AAGCCCCACTTTAGCTTTACC-3′ (reverse); Tie-2: 5′- GAGTCAGCTTGCTCCTTTATGG-3′ (forward) and 5′- AGACACAAGAGGTAGGGAATTGA-3′ (reverse).

### SWIFT-Choroid computerized quantification method

(Available to academic institutions through the authors). We developed an alternative computerized method to measure only the area in the choroidal sprouting assay covered by growing vessels. The pre-edited SWIFT-Choroid macros were copied into the plugin folder of ImageJ 1.46r (NIH) software and the appropriate shortcut for each macro was assigned. A phase contrast choroid sprouting image was opened with ImageJ and the magic wand function of the software was used to outline the choroid tissue in the center of the sprouts. The tolerance rate of the magic wand was set to 30%. The first macro deleted the choroid from the original image and read the choroid area in pixel units. The threshold function was then used to define the microvascular sprouts against the background and periphery. The free selection tools removed the background of the image. The second macro calculated the number of threshold-outlined pixels and saved an image of the selected area in the same folder as the original picture for future reference. After a group of samples was measured, the recorded measurements were copied into Microsoft excel for future analysis.

### Culture Medium

The choroidal tissue was incubated in CSC complete medium (Cell Systems, Cat. 420-500) activated with either growth factor Boost, EGM-2 medium with endothelium growth medium (EGM) kit and 5% FBS (Lonza, Cat. CC-3156) or DMEM high glucose with 10% FBS (GIBCO, Cat. 11885). All media contained 50 units/mL of Penicillin/Streptomycin (GIBCO, Cat. 15142) and 5 µg/mL of Plasmocin™ (Invitrogen, Cat. Ant-mpt).

### Trypsin digestion and removal of RPE cell layer

Adapted from previously published RPE isolation protocol[21], dissected RPE-choroid-scleral tissue was digested in 0.25% Trypsin (GIBCO, Cat. 15050-065) at 37°C for 10 to 40 minutes. After incubation, the RPE cells were removed under a microscope (ZEISS, SteREO Discovery.V8) using micro-forceps.

### Pro- and anti-angiogenic drug treatment

When treating the choroidal sprouts with VEGF (R&D system, Cat. 293-VE), the tissue was first incubated in complete medium for 48 hours and then starved in the absence of growth factors for 4 hours. Then 10 ng/ml, 50 ng/ml and 250 ng/ml of VEGF were added to growth factor reduced medium: For CSC medium a 1∶200 dilution of Boost was used instead of 1∶50 and 2.5% FBS was added to EGM-2 medium instead of 5%. When treated with anti-angiogenic factor 4-hydroxy-docosahexaenoic acid (4-HDHA) (3–15 µM; Cayman, Cat. 33200), the tissue was first incubated in complete medium for 72 hours before treatment of the samples. The vehicles of corresponding drugs (PBS for VEGF and ethanol for 4-HDHA) were used as controls.

### Statistical Analysis

Data are presented as mean ± SEM for all histograms unless otherwise indicated. Comparisons between groups were made with either 2-tail unpaired student's T-test or analysis of variance (ANOVA), followed by post-hoc Bonferroni correction for comparison among means. P<0.05 is considered statistically significant.

## Results

### Comparison and consistency of central vs. peripheral choroid sprouting

**Figure 1:**
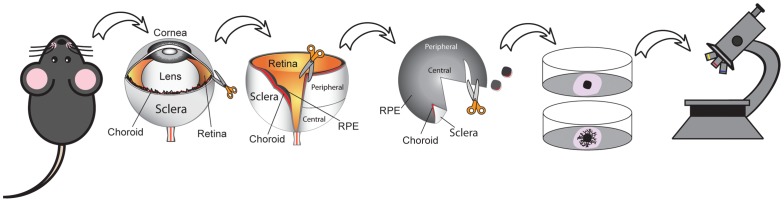
Schematic illustration of the choroid sprouting assay. Eyes are first enucleated from the mice and an initial incision is made along the corneal limbus. A micro-scissor is then used to cut 0.5 mm posterior to the corneal limbus in order to remove the cornea/iris complex and the lens. An incision perpendicular to the corneal limbus towards the optic nerve is made. After peeling off the retina from RPE/choroid/sclera complex, the central and peripheral regions of the complex are separated and further cut into approximately 1 mm×1 mm pieces and embedded in matrigel. The microvascular sprouts from the choroid tissue is visualized under a microscope.

**Figure 2:**
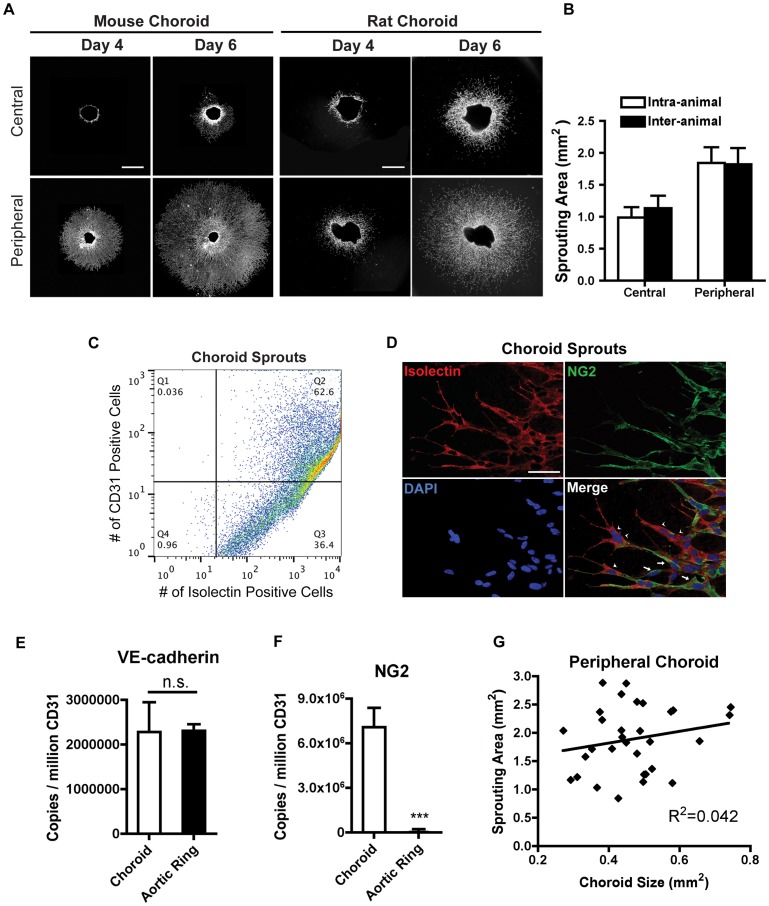
Mouse and rat central and peripheral choroid sprouting: intra- and inter-animal variability. (a) Five replicates from 5 animals were compared for the intra-animal (from the same eye of the same animal) variability and inter-animal variability of choroid sprouting. Scale bar: 500 µm. (b) The sprouting from the peripheral mouse choroid is more consistent than central mouse choroid (n = 5) (c) Flow cytometry analysis of choroid sprouting cell populations. About 60% of the cell population from the choroid sprouts stained positive for both CD-31 and isolectin, indicating ECs/macrophages. 36% of the cell population is isolectin-positive but did not stain for CD-31. (d) The extending growth cone resembles vascular tube formation *in vivo* and stains positively with isolectin GS (arrow head) surrounded by chondroitin sulfate proteoglycan neuron-glial antigen 2 (NG2) positive pericytes (arrow). Scale bar: 10 µm. (e) Real time-PCR analysis of choroid sprouts and aortic ring sprouts indicates that the expression of endothelial marker VE-cadherin is not significantly different between the two assays when normalized to CD31 expression (n = 12) p = 0.97; unpaired T-test. (f) The expression of NG2 by real time PCR is higher in choroid sprouts compared to aortic ring sprouts (n = 8) *** p<0.0001; unpaired t-test. (g) The rate of choroid sprouting is not correlated to the size of the choroid tissue embedded within the rage of 0.2 mm^2^ to 0.8 mm^2^.

We dissected the choroid attached to sclera from the retina in mouse and rat eyes, embedded fragments of the choroidal tissue in Matrigel™ and cultured them for 4–6 days (**Figure 1** **& Supporting Video S1**). Various parameters that potentially impact the reproducibility and the quantification of the assay were investigated (**Table 1**). Choroidal fragments from the central region adjacent to the optic nerve head sprouted more slowly and less consistently than those from the peripheral region adjacent to the ora serrata (**Figure 2A&B**). Therefore, choroidal tissue fragments must be obtained at the same radial distance from the optic nerve head (depending on area of interest) for consistency of the assay in both mice and rats. The mean and variation of sprouting area of the choroid samples obtained from the same eye of the same animal (intra-animal) were comparable to the sprouting area of samples from different animals (inter-animal) (**Figure 2D**), suggesting that data from inter-animal and intra-animal samples can be pooled for analysis. Interestingly, within the tested tissue punch size (i.e. 0.2 mm^2^ to 0.8 mm^2^) the size of the choroid tissue implanted did not impact the vessel-sprouting area (**Figure 2E** **& Supporting Figure S2A**). This evidence demonstrates that technical variation in choroid tissue dissection within these parameters does not cause variation in the assay.

**Table 1:** Choroid Sprouting Standardization and Optimization

**Species**	Rats	vs.	Mice
**Region of Choroid**	Central	vs.	Peripheral
**Normalization**	Absolute Area	vs.	Relative to Control
**Quantification**	Manual	vs.	SWIFT
**Impact of RPE**	With RPE	vs.	Without RPE
**Medium**	EC selective	vs.	Non-selective
**Age**	Young	vs.	Old
**Strain**	129S6	vs.	C57BL/6J
**Drug Treatment**	Pro-angiogenic	vs.	Anti-angiogenic

### Sprouts consist of ECs, pericytes and monocytes/macrophages

FACS cell sorting analysis indicated that approximately 60% of the cell populations of the sprouts stain positively with anti-CD31 antibody (**Figure 2C** **& Supporting Figure S1A**). CD31 serves as a biomarker of ECs but may also be expressed in some macrophages. The vascular sprouts from the choroid were tube-like growth cones of ECs (positive for isolectin GS) surrounded by pericytes (positive for chondroitin sulfate proteoglycan neuron-glial antigen 2 (NG2)) (**Figure 2D**). Compared to the aortic ring assay, the choroidal sprouts expressed similar quantities of endothelial markers (i.e. VE-cadherin and Tie-2) when normalized to CD31 (**Figure 2E** **& Supporting Figure S1D)**. Higher mRNA expression of NG2, a microvascular morphogenesis marker, was detected in choroidal sprouts compared to aortic ring sprouts (**Figure 2F**), indicating that the choroidal sprouts were indeed microvessels. A population of isolectin and CD68-positive cells was also detected in the sprouts adjacent to planted choroidal tissue, but not at the tips of the sprouts. These isolectin and CD68-positive cells exhibited monocyte/macrophage-like cell morphology (**Supporting Figure S1B**). Compared to aortic ring sprouts, the expression of CD68 was much higher in the choroidal sprouts (**Supporting Figure S1E)**. The latter observations were consistent with what appears to occur in choroidal neovascularization *in vivo* [22]. Aortic ring sprouts were less differentiated and contained cells positive for both EC specific stain, Isolectin and pericyte marker, Desmin (**Supporting Figure S1C**). Importantly, the choroidal sprouting area was not affected by the size of the choroidal tissue implanted (between 0.2 mm^2^ to 0.8 mm^2^) (**Figure 2G** **& Supporting Figure S2A**).

### The choroid sprouting assay is highly reproducible

**Figure 3:**
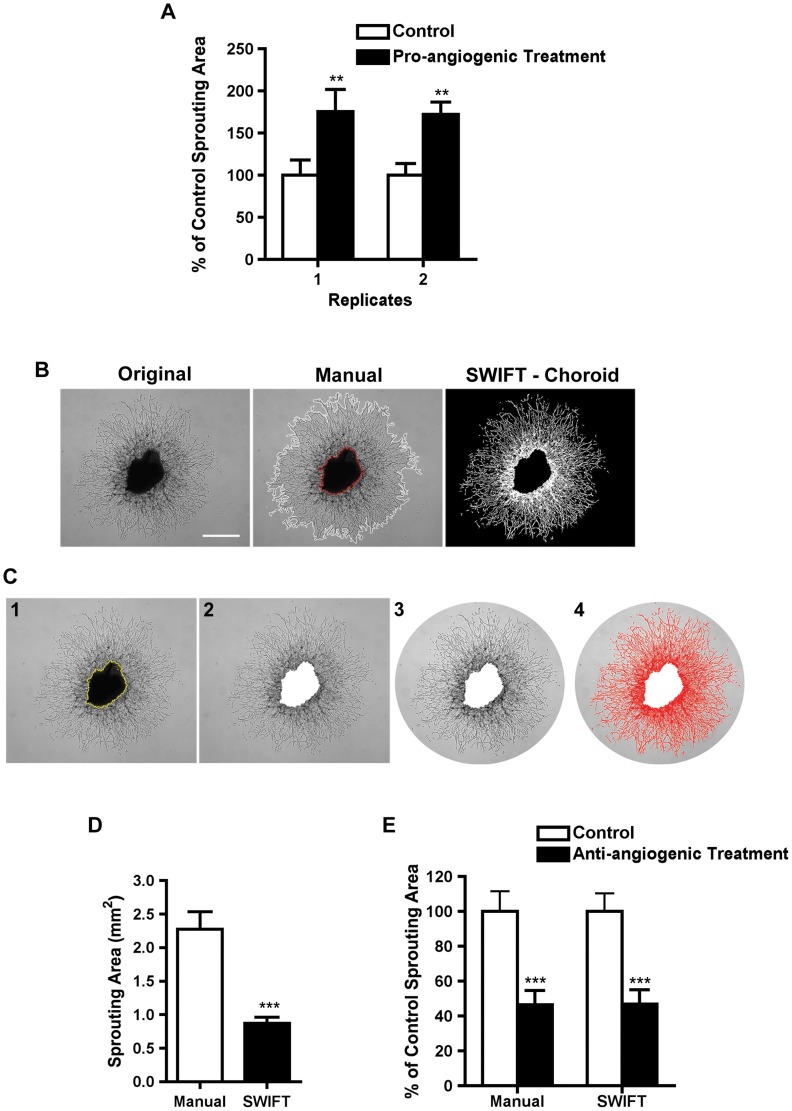
Computerized and manual choroid assay quantification methods yield reproducible and comparable normalized results. (a) The percentage change between control and treatment groups was highly reproducible between independent experiments run at different times; (n = 6–12) ** p = 0.0042; 2-way ANOVA with Bonferroni correction; (b) Representative photos demonstrate manual and computerized quantification of choroid endothelial sprouts. Scale bar: 500 µm. (c) A step-by-step demonstration of SWIFT-Choroid method quantifying the area of the sprouts. (c1) The area of the choroid tissue was first selected by the magic wand tool from the ImageJ software. (c2) Step 1 of the macro was then used to delete the choroid tissue from the image and calculate the choroid area in pixels. (c3) The excess area of the image was then removed using the polygon selection tool and (c4) the threshold function and the second step of the macro was followed to calculate the sprouting area in pixels. (d) The SWIFT-Choroid method calculates sprouting area without extracellular space. The absolute value of the calculated area is 2.27±0.26 mm2 for manual (n = 11) versus 0.87±0.09 mm2 (n = 11) *** p<0.0001; unpaired t-test. (e) However, the differences between control (n = 11) and treated groups normalized to respective controls (n = 12) were similar for each method: 46.6±8.1% using manual quantification and 46.9±8.1% using SWIFT-Choroid. *** p<0.0001 between treatment group and control group, p = 0.986 between quantification methods; 2-way ANOVA.

Traditionally, the sprouting area of endothelial organotypic culture is quantified by subtracting the area of the tissue explant from the total area occupied by the sprouts [10], [23]. The percentage change in sprouting areas in response to angiogenic stimuli compared to controls was highly reproducible between replicates (175±26% vs. 172±14%) (**Figure 3A**). However, absolute sprouting areas between independent experiments were slightly different (**Supporting Figure S2C**), reflecting the slight natural variation of tissue preparation and culture conditions from experiment to experiment, and indicating the importance of normalizing to control within each experiment. Because the sprouting area did not correlate with the size of the choroid tissue implanted (**Figure 2E** **& Supporting Figure S2A**), we confirmed that the area of the explanted tissue should be subtracted from the sprouting area [10], [23] and should be normalized to the control groups within the same experimental set. The area of explanted tissue should not be used to normalize the sprouting area. (Because the data points were normally distributed, we used the Student's T-test and Analysis of Variance (ANOVA) to examine the statistical significance between groups (**Supporting Fig. S2D**).

### Standardized quantification of sprouting (Macros available from the authors)

(Macros available from the authors.) To increase the efficiency and accuracy of the quantification we developed a computerized SWIFT-Choroid method that uses pre-installed macros for ImageJ software to calculate the area of fine vascular sprouts without including the space between vessels observed with manual quantification (**Figure 3B & C**). Hence, the absolute reading differed between the manual (including the extracellular space) and SWIFT quantification methods (excluding the extracellular space; **Figure 3D**). After normalization of each treatment group to the control group, however, the results from the two quantification methods were highly consistent (46.6±8.1% using manual quantification and 46.9±8.1% using SWIFT-Choroid) (**Figure 3E**). As expected, the SWIFT-Choroid computer-image analysis method was ∼10 times faster than the manual approach (∼2 minutes versus ∼20 minutes per photo using manual quantification).

### Influence of retinal pigment epithelium (RPE) on the choroid sprouting assay

#### A. RPE potentiates endothelial sprouting in endothelium specific media

Retinal pigment epithelium (RPE) is a single layer of pigmented cells lying between the photoreceptors of the retina and choriocapillary bed. It plays a crucial role in maintaining the visual circle by phagocytizing the outer segment of the photoreceptors and converting all-trans-retinal to 11-cis-retinal. The RPE and choroidal vessels are known to interact such that the RPE exerts both pro- and anti-angiogenic effects [24] by releasing both pro- and anti- angiogenic factors like vascular endothelial growth factor (VEGF) and pigment epithelial derived factor (PEDF) [25]. Choroidal tissue with or without the RPE attached can be used in the choroid sprouting assay. We compared the sprouting of the choroid with and without RPE in different media, and with trypsin removal of RPE to assess the effect of trypsin on the assay.

**Figure 4:**
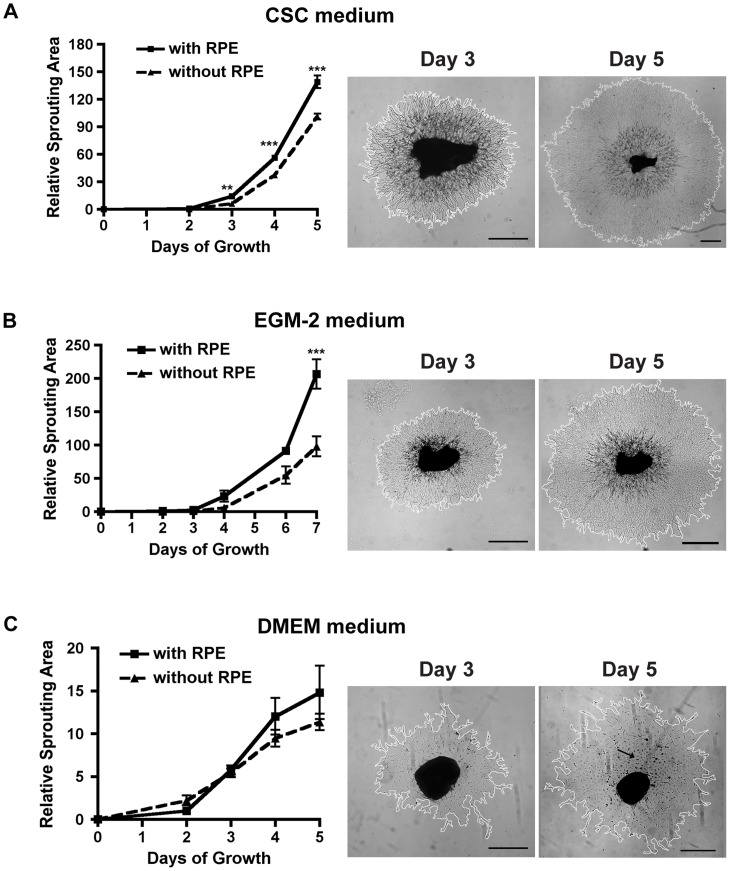
RPE cells promote choroidal endothelial sprouting in endothelial-selective media. Three different cell culture media (CSC, EGM-2 and DMEM) were compared for standardizing choroidal tissue response. (A) Complete CSC medium with growth factor Boost promotes rapid sprouting. An intact RPE layer on choroid further accelerates sprouting (n = 5–12) p<0.0001 compared to choroid without RPE. (B) RPE cells also potentiate choroid sprouting in EGM-2 medium (n = 6 for each time point) p<0.0001, (C) However, in DMEM there is no difference in sprouting rate (n = 6–18, p = 0.1) regardless of the presence of RPE. The sprouting in DMEM medium contained RPE-like contamination (arrows) and the sprouts did not form growth cones as shown in CSC or EGM-2 medium. All comparisons are 2-way ANOVA with Bonferroni correction. Scale bar: 500 µm.

Sprouting from choroidal tissue without RPE was significantly slower in EC specific media such as CSC medium (**Figure 4A**) and EGM-2 medium (**Figure 4B**), but not in non-selective cell medium such as DMEM medium (**Figure 4C**). Therefore, the RPE-choroid co-explant assay can be used to analyze the RPE-choroid interaction and its response under different conditions. For instance, to understand the impact of a drug on choroid vessels alone, one may use choroidal tissue with the RPE removed to conduct the sprouting assay. In another case, if one is interested in the impact of the RPE-derived factors on choroid angiogenesis, conditional gene knockout in RPE cells may be studied in choroid explants with the RPE left in place. The sprouting in DMEM medium did not display tube-like structures and contained RPE-like cellular debris adjacent to the explanted tissue (**Figure 4C**). These results suggest DMEM may promote RPE proliferation and may not be ideal to support vascular cell interactions that give rise to vascular tubes.

#### B. Trypsin (used to remove RPE) does not influence the choroid sprouting assay

To completely remove the RPE layer, RPE-choroid tissue may be treated with trypsin to dissociate RPE from choroid before explanting. Since trypsin could activate pro-angiogenic receptors and promote vascular proliferation [26], the potential angiogenic effect of trypsin was examined in the choroid sprouting assay. After 10–40 minutes of incubation, no appreciable difference was detected with or without trypsinization, demonstrating that 20 minutes of trypsin treatment per se does not significantly affect choroid sprouting (**Supporting Figure S3**). A short incubation time of 10 minutes incompletely dissociates RPE and therefore induces high variation in sprouting rate, whereas a longer incubation of 40 minutes changes the texture of the choroidal tissue. Hence, a 20-minute incubation time is recommended if trypsinization is necessary.

### Choroid tissue from aging animals sprouts more slowly than from young animals

**Figure 5:**
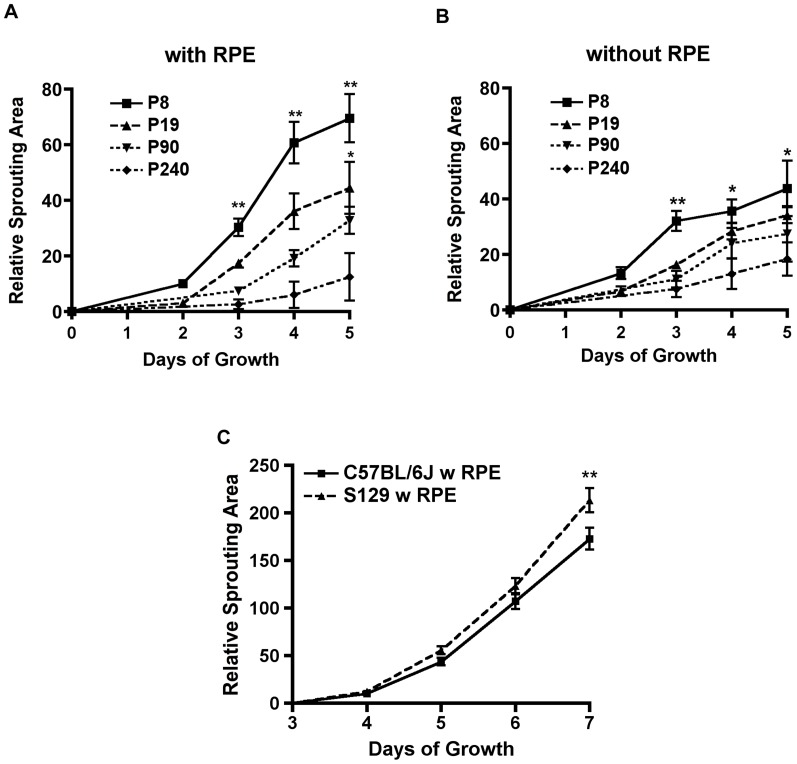
The age and strain of the animal affects the rate of choroidal sprouting in explant culture. (A&B) Choroid with or without RPE from P8 animals sprouts significantly faster than that from P240 aging animals (n = 6–18, p<0.0001 with RPE; n = 6–12, p = 0.0002 without RPE). (C) The choroid explants from 129S6/SvEvTac mice (n = 10) grow significantly faster than choroid explants from C57BL/6J mice (n = 12) at day 7 p = 0.0017. All comparisons are 2-way ANOVA with Bonferroni corrections.

Choroidal vascular degeneration and later-stage central choroidal neovascularization are prominent features of AMD [27]. To assess the influence of aging on choroidal endothelial proliferation, the choroidal tissue from young and aged animals was isolated. The choroid from older animals (postnatal day (P) 240, i.e. 8 months old) sprouted more slowly than choroid from P8 pups. This age-dependent difference in sprouting was RPE independent (12.4±8.5 vs. 69.5±8.6 with RPE and 18.3±6.0 vs. 43.7±7.1 without RPE) (**Figure 5A & B**). Previous studies in neonatal rats indicate that the choroid vascular bed continues to grow during the first two weeks of postnatal development [**10**], which rationalizes why the choroid from P8 mice sprouts faster than choroid tissue from P19 mice. Since aging in general suppresses the proliferative capacity of cells, the findings here may suggest a decreased ability of reparative angiogenesis in aging choroidal endothelium.

### Strain difference: choroidal tissue from 129S6/ScEvTac mice sprouts faster than that from C57BL/6J mice

The normal retinal vascular development in various mouse strains varies significantly [28]. We investigated the choroid sprouting response of 129S6/SvEvTac mice compared to C57BL/6J mice. After 7 days of incubation, the choroid explants from 129S6/SvEvTac animal grew significantly faster than choroid obtained from C57BL/6J mice (213.3±12.7 vs. 172.9±11.3 respectively; **Figure 5C**).

### Pharmacological intervention in the choroid sprouting assay is reproducible

**Figure 6:**
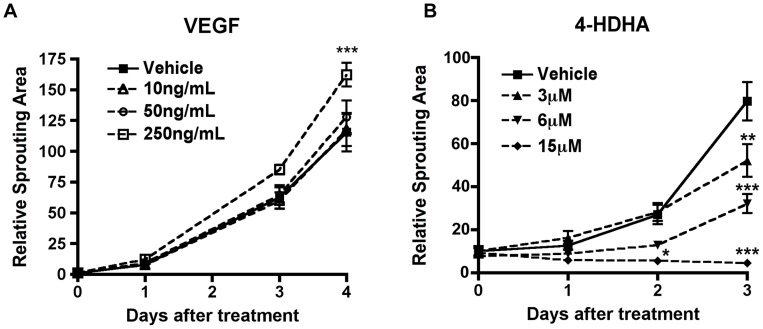
Choroid sprouting assay responds dose-dependently to pro- and anti-angiogenic factors. (A) Choroid sprouting increases with increasing doses of VEGF (n = 8 for each time point). After 4 days of incubation, 250 ng/mL of VEGF increased the sprouting compared to vehicle treated control *** p<0.001. (B) After treating with 4-HDHA, the sprouting rate decreased as the concentration of 4-HDHA increases. At 15 µM 4-HDHA, the existing sprouts regressed over time (n = 4–8 for each treatment group) p<0.0001 between different 4-HDHA doses. All comparisons are 2-way ANOVA with Bonferroni correction.

VEGF is a well-studied pro-angiogenic molecule [29]. In addition, our previous study indicated that 4-hydroxy-docosahexaenoic acid (4-HDHA), a lipid metabolite of dietary ω-3 polyunsaturated fatty acids (PUFAs), inhibits EC proliferation in both the spheroid and aortic ring assays [30]. Here we tested VEGF and 4-HDHA in the choroid sprouting assay. VEGF promoted sprouting in the choroid assay in a dose-dependent manner (**Figure 6A**). Furthermore, 4-HDHA reproducibly and dose-dependently suppressed choroid sprouting (**Figure 6B**), suggesting that the choroid sprouting assay can be used to assess the angiogenic potential of pharmacologic compounds.

## Discussion

Here we report the development and standardization of an organotypic microvascular sprouting assay that can be obtained from the choroid of transgenic mice to evaluate the effect of genetic manipulation on microvascular proliferation. This assay is highly reproducible and pertinent to angiogenesis research, particularly in the neovascular AMD field. Additionally, we evaluated the effects of age, region of the choroid and specific endothelial growth media on the assay. The choroid sprouting assay can be used to evaluate pro- and anti-angiogenic pharmacological interventions. Importantly, the choroid sprouting assay allows for analysis of the interaction between choroidal endothelial cells and their adjacent cells (e.g. RPE cells) to uncover mechanisms that control choroidal vascular growth of specific relevance (but not exclusively) to sub-retinal proliferative disorders. We also described a reproducible and semi-automatic method that standardizes quantitative analysis of choroidal sprouting area.

In this study, we discovered that different culture media affects the outcome of the choroid sprouting assay. In previous *ex vivo* assays, DMEM was used [31]. However, we found that DMEM is not formulated for optimal EC growth as it promotes proliferation of other types of cells (such as RPE), which influence vascular proliferation (**Figure 4C**). Therefore DMEM is unsuitable for the choroid sprouting assay. Various EC specific culture media have been suggested for *ex vivo* vascular cultures, including MCDB 131 medium [32] and EGM-2 medium [30], [33]. CSC complete medium is a modified and optimized DMEM/F12 (1∶1) medium containing 10% of serum by volume and endothelial growth factor boost, which is designed for primary ECs and provided the optimum sprouting for the choroid explants.

Our results also show that RPE cells promote choroidal vascular proliferation under some specific culture medium conditions, consistent with the proliferative effects of RPE on EC spheroids grown on a monolayer of RPE cells [19]. Hence, the assay we describe (with and without RPE) provides another tool to study RPE-choroidal interaction particularly in AMD studies. Since choroidal involution and central choroidal neovascularization are associated with AMD and aging [34], we investigated the impact of age on choroidal vascular proliferation. Aging choroid sprouts more slowly than choroid from newborn and juvenile mice. This phenomenon is consistent with choroidal involution seen in the dry form of AMD with geographic atrophy [27]. Among AMD patients, 85–90% have dry AMD [35]. The choroid sprouting assay may be a versatile tool to facilitate investigations on mechanisms underlying AMD pathogenesis.

An *ex vivo* incubation of choroidal tissue was first described by Kobayashi et al. [31], [36]–[38]. These studies used isolated choroid to test pharmacological interventions with the potential to treat DR and AMD. The assay was quantified by counting the number of vessels. We have characterized the choroid assay in more detail and standardized the quantification method. In our study, we found that the growth rates of replicates normalized to controls within an experiment are highly consistent (**Figure 2b**) and comparisons between normalized, treated groups in separate experiments are very reproducible (**Figure 3a**).

This assay of microvascular angiogenesis is likely to be a useful addition to other assays. Culture of pure vascular ECs *in vitro* is limited as a microvascular assay in large part because of the difficulty in isolating mouse ECs, which is important for evaluating the impact of genetic manipulation on angiogenesis of transgenic mice. Although useful as an assay examining effects of interventions on EC biology, cultured primary ECs may undergo phenotypic drifts, including changes in ion channel expression and function, calcium homeostasis, as well as ECs specific protein expression [20], [39]. Such heterogeneity is affected by the autocrine action of growth factors, hormones and vasoactive substances [40], [41] and explains in part the variability and occasional inconsistencies reported with these cells. Furthermore, in the absence of their interactive partner cells and extracellular matrix *in vitro* cultures of ECs may lose many of their physiological properties, such as the ability to form vascular tubes [42]. The aortic ring assay is very useful to assess sprouts from large vessels from mice but may not accurately reflect a tissue-specific microvascular response [43].

In summary, this study characterized and standardized a highly reproducible, efficient and quantifiable choroid microvascular *ex vivo* sprouting assay that can be obtained from rat and transgenic mice to study microvascular angiogenesis (**Table 2**). This method provides a new experimental tool not only for AMD studies, but also for physiological and pharmacological research related to microvascular diseases in general.

**Table 2:** Suggested Conditions for Choroid Microvascular Sprouting Assay

**Region of Choroid**	Peripheral choroid
**Normalization**	Relative to control within the same batch
**Quantification**	SWIFT-Choroid semi-automated quantification
**Trypsinization**	No trypsinization if possible or 20 min 0.25% Trypsin at 37°C
**Medium**	EC selective media
**Age**	P20 for optimum growth rate
**Strain**	Consistent background between wild type and transgenic animals

## Supporting Information

Figure S1 (A) Flow cytometry analysis of sprouts with R-phycoerythrin (PE) labeled anti-CD31 antibody confirms that over half of the cell population is CD31 positive. (B) Adjacent to choroid tissue, a small population of CD68 and isolectin positive cells was detected. These cells also exhibit the morphology of monocytes/macrophages. (C) The cells composing aortic ring sprouts are less differentiated and express both EC marker Isolectin and Desmin. (D) Real time-PCR analysis of choroid sprouts and aortic ring sprouts indicates that the expression of endothelial marker Tie-2 is not significantly different between the sprouts of two assays (n = 8) p = 0.143; unpaired T-test. (E) The expression of CD68 tested by real time PCR is significantly higher in choroid sprouts compared to aortic ring sprouts indicating that there is a larger macrophage population present in the choroid assay comparing to the aortic ring assay (n = 12) *** p<0.0001; unpaired t-test.
(TIF)

Figure S2 (A) The rate of choroid sprouting is not correlated with the size of the choroid tissue embedded using choroid from the central regions of the eye. (B) Impact of initial sprouting rate. (C) The absolute choroid sprouting areas in mm^2^ between independent experiments could be significantly different (two independent experiments conducted 2 days apart) (n = 11 for control and n = 6 for treatment) ** p = 0.0058; unpaired t-test. (D) Samples from the control group and the treated groups are normally distributed indicating that statistical analysis that assumes normal distribution of the samples can be used in this study.
(TIF)

Figure S3 Trypsin, used to remove RPE, does not affect choroid sprouting (n = 3 for each time point) p = 0.1225 between different groups, 2-way ANOVA with Bonferroni correction. However, a 10 min incubation incompletely dissociates RPE, causing incomplete removal of RPE which induces high variation in sprouting rate, whereas long-term incubation (i.e. 40 min) changes the texture of choroid tissue. Hence, 20 min incubation is recommended for trypsinization.
(TIF)

Video S1 Demonstration of isolation of choroid from the mouse eye.
(MOV)
