# Correction: Choroid Sprouting Assay: An *Ex Vivo* Model of Microvascular Angiogenesis

**DOI:** 10.1371/annotation/c6b85ec4-996d-4daf-863a-44260a888470

**Published:** 2013-08-13

**Authors:** Zhuo Shao, Mollie Friedlander, Christian G. Hurst, Zhenghao Cui, Dorothy T. Pei, Lucy P. Evans, Aimee M. Juan, Houda Tahiri, François Duhamel, Jing Chen, Przemyslaw Sapieha, Sylvain Chemtob, Jean-Sébastien Joyal, Lois E. H. Smith

The name of the eighth author was spelled incorrectly. The correct name is: Houda Tahiri. 

