# Supplementary figures and images for: Choroid Sprouting Assay: An Ex Vivo Model of Microvascular Angiogenesis
Source: PLoS One. 2013 Jul 26;8(7):e69552. doi: 10.1371/journal.pone.0069552 (PMC3724908; doi:10.1371/journal.pone.0069552)

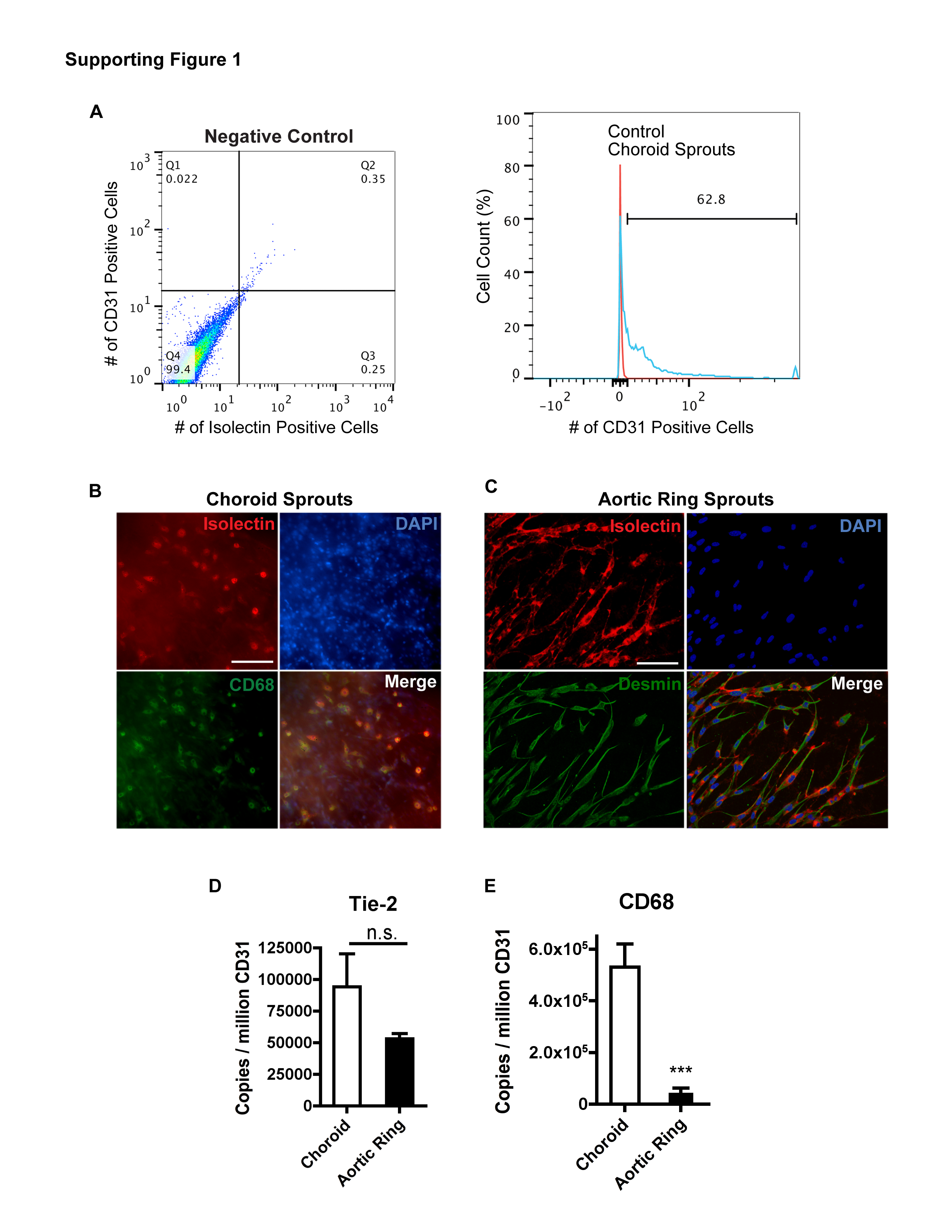

Supplement: Figure S1 — (A) Flow cytometry analysis of sprouts with R-phycoerythrin (PE) labeled anti-CD31 antibody confirms that over half of the cell population is CD31 positive. (B) Adjacent to choroid tissue, a small population of CD68 and isolectin positive cells was detected. These cells also exhibit the morphology of monocytes/macrophages. (C) The cells composing aortic ring sprouts are less differentiated and express both EC marker Isolectin and Desmin. (D) Real time-PCR analysis of choroid sprouts and aortic ring sprouts indicates that the expression of endothelial marker Tie-2 is not significantly different between the sprouts of two assays (n = 8) p = 0.143; unpaired T-test. (E) The expression of CD68 tested by real time PCR is significantly higher in choroid sprouts compared to aortic ring sprouts indicating that there is a larger macrophage population present in the choroid assay comparing to the aortic ring assay (n = 12) *** p<0.0001; unpaired t-test. (TIF) [file pone.0069552.s001.tif]

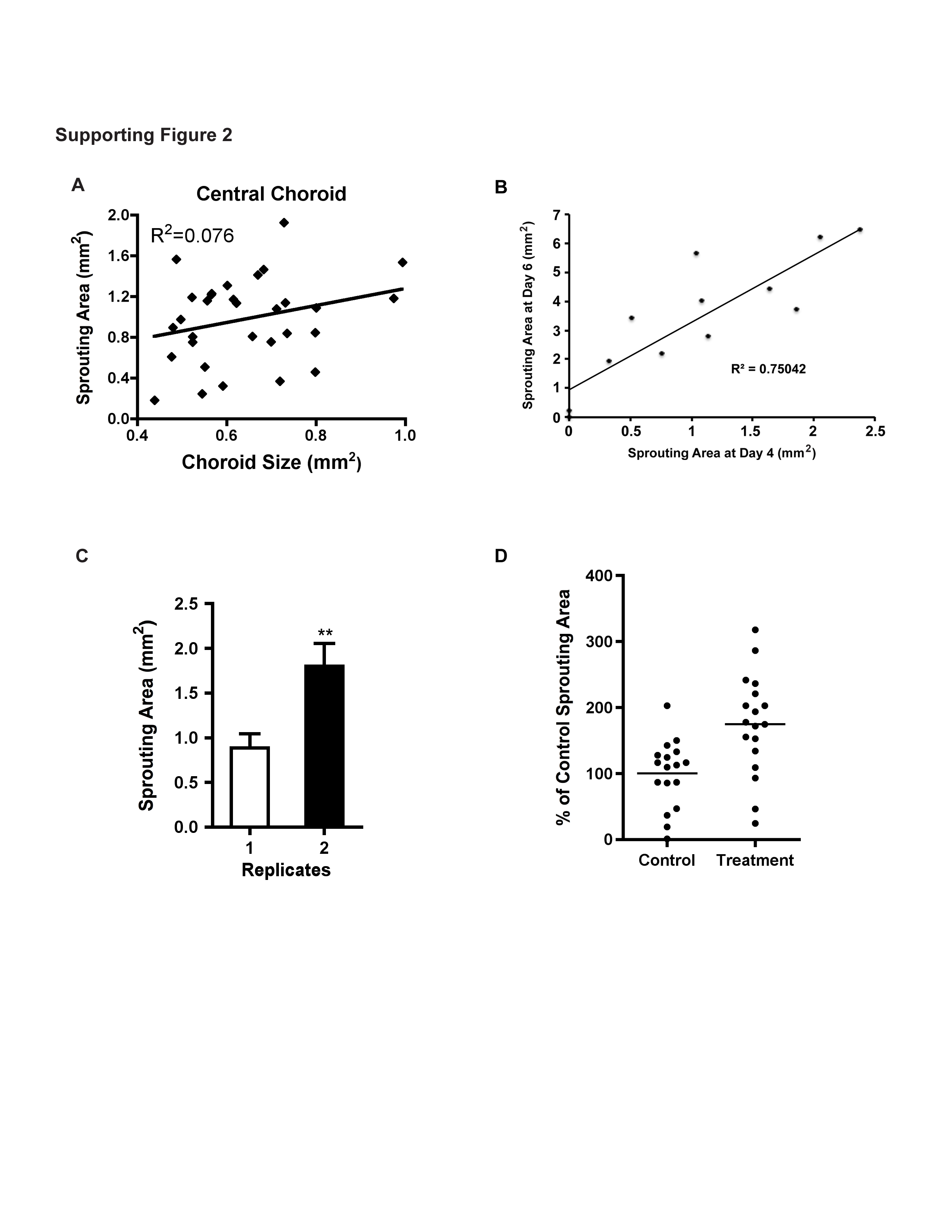

Supplement: Figure S2 — (A) The rate of choroid sprouting is not correlated with the size of the choroid tissue embedded using choroid from the central regions of the eye. (B) Impact of initial sprouting rate. (C) The absolute choroid sprouting areas in mm2 between independent experiments could be significantly different (two independent experiments conducted 2 days apart) (n = 11 for control and n = 6 for treatment) ** p = 0.0058; unpaired t-test. (D) Samples from the control group and the treated groups are normally distributed indicating that statistical analysis that assumes normal distribution of the samples can be used in this study. (TIF) [file pone.0069552.s002.tif]

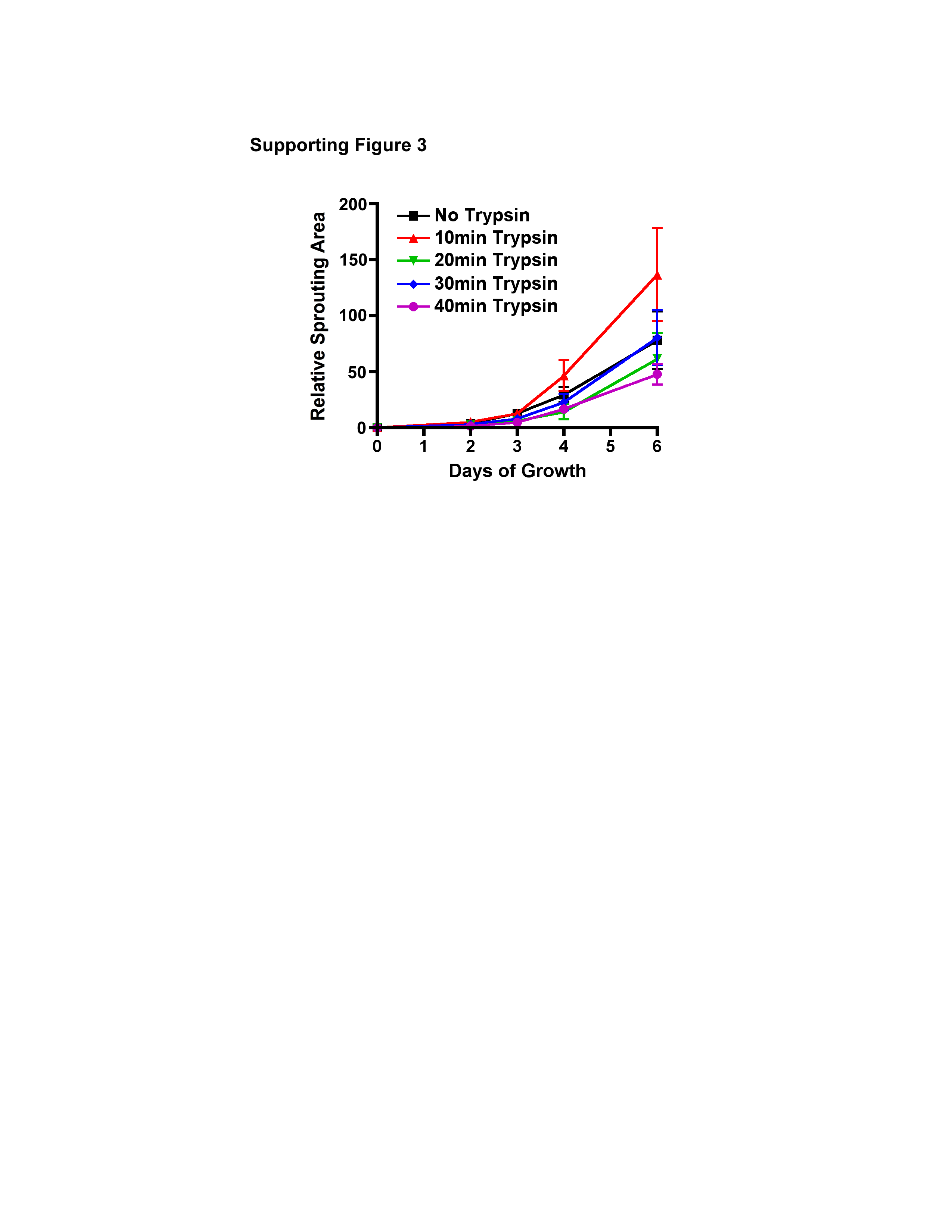

Supplement: Figure S3 — Trypsin, used to remove RPE, does not affect choroid sprouting (n = 3 for each time point) p = 0.1225 between different groups, 2-way ANOVA with Bonferroni correction. However, a 10 min incubation incompletely dissociates RPE, causing incomplete removal of RPE which induces high variation in sprouting rate, whereas long-term incubation (i.e. 40 min) changes the texture of choroid tissue. Hence, 20 min incubation is recommended for trypsinization. (TIF) [file pone.0069552.s003.tif]
